# Emotion Recognition in People With Dementia and Caregiver Affect During Dyadic Interactions

**DOI:** 10.1093/geroni/igaf122.291

**Published:** 2025-12-31

**Authors:** Casey Brown, Yuxuan Chen, Robert Levenson

**Affiliations:** Georgetown University, Washington, District of Columbia, United States; Stanford University, Stanford, California, United States; University of California, Berkeley, Berkeley, California, United States

## Abstract

Dementia caregivers may experience negative emotions during interactions with their care recipients, yet little research has explored the specific factors that contribute to this response in a dyadic context. Based on research demonstrating care recipient’s emotion recognition impairments are linked with their caregivers’ depressive symptoms, we hypothesized that impairments in care recipient’s emotion recognition relates to increases in their caregiver’s negative affect during interactions with their care recipient. A sample of 100 caregiver-care recipient dyads participated in the study. Caregivers reported on their care recipients’ emotion recognition using a well-validated measure. Dyads engaged in a 10-minute conflict-based conversation in a laboratory setting, after which caregivers watched video recordings of their conversation and continuously rated their emotional valence using a rating dial. These ratings were used to assess changes in caregivers’ affect over the course of the conversation. Caregivers whose care recipients had greater deficits in emotion recognition experienced more pronounced increases in negative affect throughout the conversation, even after accounting for caregivers’ baseline affect, sex, and age, as well as care recipients’ diagnosis, level of cognitive impairment, neuropsychiatric symptoms, and functional limitations. These results demonstrate an association between care recipients’ emotion recognition skills and caregivers’ emotional experiences. When care recipients struggle to recognize emotions, caregivers may be more likely to experience worsening negative affect during interactions, highlighting a potential target for caregiver support interventions.

